# Multimorbidity and Blood Pressure Control in Patients Attending Primary Care in Canada

**DOI:** 10.1177/21501319231215025

**Published:** 2023-12-14

**Authors:** Tu N. Nguyen, Sumeet Kalia, Peter Hanlon, Bhautesh D. Jani, Barbara I. Nicholl, Chelsea D. Christie, Babak Aliarzadeh, Rahim Moineddin, Christopher Harrison, Clara Chow, Martin Fortin, Frances S. Mair, Michelle Greiver

**Affiliations:** 1 University of Sydney, Sydney, NSW, Australia; 2 University of Toronto, Toronto, ON, Canada; 3 University of Glasgow, Glasgow, UK; 4 Université de Sherbrooke, Saguenay, QC, Canada; 5 North York General Hospital, Toronto, ON, Canada

**Keywords:** hypertension, high blood pressure, blood pressure control, multimorbidity, primary care

## Abstract

**Background:**

There has been conflicting evidence on the association between multimorbidity and blood pressure (BP) control. This study aimed to investigate this associations in people with hypertension attending primary care in Canada, and to assess whether individual long-term conditions are associated with BP control.

**Methods:**

This was a cross-sectional study in people with hypertension attending primary care in Toronto between January 1, 2017 and December 31, 2019. Uncontrolled BP was defined as systolic BP ≥ 140 mmHg or diastolic BP ≥ 90 mmHg. A list of 11 a priori selected chronic conditions was used to define multimorbidity. Multimorbidity was defined as having ≥1 long-term condition in addition to hypertension. Logistic regression models were used to estimate the association between multimorbidity (or individual long-term conditions) with uncontrolled BP.

**Results:**

A total of 67 385 patients with hypertension were included. They had a mean age of 70, 53.1% were female, 80.6% had multimorbidity, and 35.7% had uncontrolled BP. Patients with multimorbidity had lower odds of uncontrolled BP than those without multimorbidity (adjusted OR = 0.72, 95% CI 0.68-0.76). Among the long-term conditions, diabetes (aOR = 0.73, 95%CI 0.70-0.77), heart failure (aOR = 0.81, 95%CI 0.73-0.91), ischemic heart disease (aOR = 0.74, 95%CI 0.69-0.79), schizophrenia (aOR = 0.79, 95%CI 0.65-0.97), depression/anxiety (aOR = 0.91, 95%CI 0.86-0.95), dementia (aOR = 0.87, 95%CI 0.80-0.95), and osteoarthritis (aOR = 0.89, 95%CI 0.85-0.93) were associated with a lower likelihood of uncontrolled BP.

**Conclusion:**

We found that multimorbidity was associated with better BP control. Several conditions were associated with better control, including diabetes, heart failure, ischemic heart disease, schizophrenia, depression/anxiety, dementia, and osteoarthritis.

## Introduction

Hypertension is a risk factor for cardiovascular disease and other chronic health conditions. The prevalence of hypertension increases with age, with a prevalence of only 27% in people younger than 60 years, but 74% in people aged 80 years and older.^1^ Globally, blood pressure (BP) control is still suboptimal, with control rates of approximately 20% for people with hypertension.^2^ Poor BP control can increase the risk of heart failure, coronary heart disease, peripheral artery disease, renal failure, stroke, and dementia, and therefore, increase the risk of developing multimorbidity in people with hypertension.^3^ Multimorbidity, defined as having 2 or more chronic conditions, has become a primary healthcare concern.^4,5^

Two out of 3 people with hypertension have additional long-term condition.^6^ A systematic review of 45 studies showed that the overall prevalence of multimorbidity was 66.1% (when multimorbidity was defined as having ≥2 chronic conditions), and 44.2% (when multimorbidity was defined as having ≥3 chronic conditions) in older adults in high-income countries.^7^ In the United States, 81 million adults were estimated to have multimorbidity in 2020.^8^ In Canada, the prevalence of multimorbidity is also high, with a reported prevalence ranging from around 30% to 70% in primary care settings, and around 17% to 59% in the general population.^9^ Multimorbidity increases healthcare costs and healthcare utilization including hospitalizations.^10,11^

Several studies have examined the impact of multimorbidity on BP control, and there has been conflicting evidence on the association between multimorbidity and hypertension control.^12-17^ While some studies show that the presence of multimorbidity was positively associated with BP control,^12,13,16^ other studies suggested that people with more comorbidities had poorer management and control of hypertension.^15,17^ There is minimal evidence of this association in the Canadian population. Therefore, this study aimed to investigate the association between multimorbidity and BP control in patients attending primary care in Canada, and to assess if individual comorbidities are associated with BP control.

## Methods

This study used data from the University of Toronto Practice-Based Research Network (UTOPIAN), a primary care electronic medical records (EMRs) database. The UTOPIAN database contains de-identified records from participating family medicine clinics in Ontario, Canada, with most providers practicing in the Greater Toronto Area.^18^ Data include de-identified patient-level information on various factors including demographics, medical diagnoses, procedures, medications, immunizations, laboratory test results, vital signs, risk factors, and clinical notes.

### Study Design and Eligible Patients

**Figure 1.**
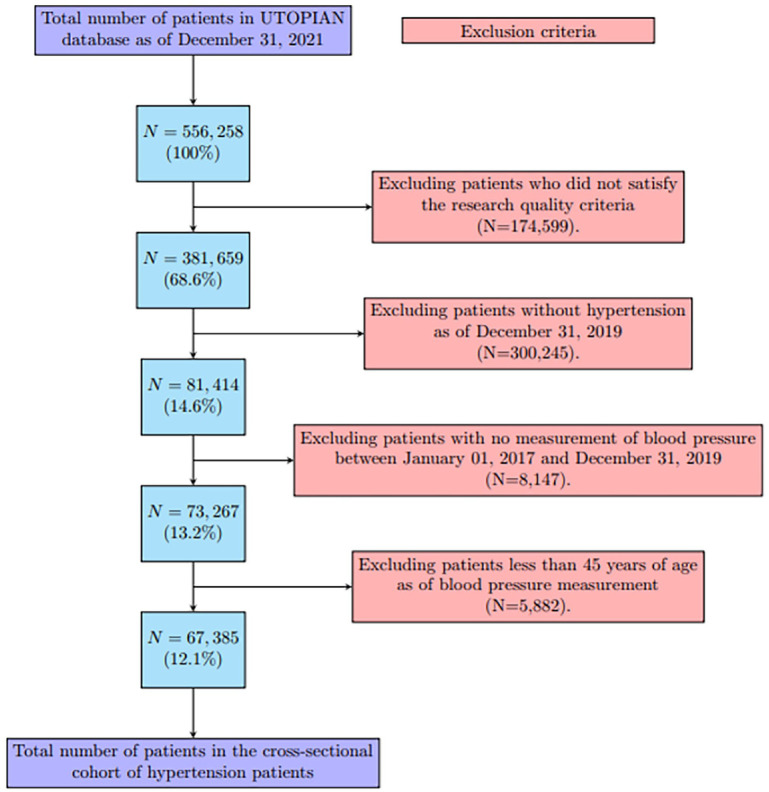
Flowchart for the generation of this cross-sectional cohort.

This is a cross-sectional study using the UTOPIAN 2021Q4 database with a start date of January 1, 2017 and an end date of December 31, 2019. This end date was chosen to avoid the COVID-19 pandemic-related effects on the data. We identified a cohort of patients who met the following selection criteria; had hypertension before December 31, 2019; had blood pressure recorded between January 1, 2017 and December 31, 2019; and were at least 45 years old when their blood pressure was measured. Figure 1 provides a flowchart for the cross-sectional cohort.

We defined the hypertension phenotype using the following criteria (Supplemental Appendix 2):

1. Free text documentation of hypertension was found within the (past or present) health condition section of the cumulative patient profile, including the following terms: hypertension, hypertensive, htn; OR
2. Anti-hypertensive medication was prescribed and an elevated blood pressure reading was recorded at any point in the EMR (elevated blood pressure reading is defined as systolic blood pressure ≥140 mmHg or diastolic blood pressure ≥90 mmHg); OR
3. Anti-hypertensive medication was prescribed and a billing record with the diagnosis code for hypertension (ICD-9 code 401) was recorded at any point in the EMR; OR
4. A billing record with the diagnosis code for hypertension (ICD-9 code 401) and an elevated blood pressure reading was recorded at any point in the EMR (elevated blood pressure reading is defined as systolic blood pressure ≥140 mmHg or diastolic blood pressure ≥90 mmHg)

### Multimorbidity

Multimorbidity was defined as having 1 or more long-term conditions in addition to hypertension. This definition of multimorbidity is similar to definitions used in other studies (REFs).^12,19,20^ A list of 11 a priori selected chronic conditions was used to define multimorbidity, in alignment with the Centers for Disease Control and Prevention (CDC) recommendations for defining and measuring multiple chronic conditions when using Medicare claims.^21^ These long-term condition are: ischemic heart disease, heart failure, atrial fibrillation, diabetes, chronic obstructive pulmonary disease, chronic kidney disease, cancers, osteoarthritis, dementia, depression and/or anxiety, and schizophrenia.

### Covariate Definitions

We used the most recent information on Body Mass Index (BMI) and smoking status as of December 31, 2019. BMI was grouped into underweight (≤18.4), normal (18.5-24.9), overweight (25.0-29.9), obese class I (30.0-34.9), obese class II (35.0-39.9), and obese class III (≥40.0). We calculated age (in years) on the date when the blood pressure measurement was recorded between January 1, 2017 and December 31, 2019. We used rurality and neighborhood income quintiles (as an indicator of socioeconomic status) using data from the 2016 Statistics Canada Census.^22^

We identified different classes of hypertension medication (including angiotensin converting enzyme inhibitors (ACEi), angiotensin II receptor blocker (ARBi), calcium channels, beta-blockers, diuretics, and other classes of hypertension medication) that were prescribed during the 6 = months prior to the blood pressure measurement. The full description of drug names for each class of hypertension medication is provided in Supplemental Appendix section (i), along with the algorithm used to determine the presence of hypertension in EMRs (see Supplemental Appendix section (ii)).

### Outcome Measure

The primary outcome measurement was the most recent BP measure recorded during the study period (January 01, 2017-December 31, 2019). Uncontrolled BP was defined as having either systolic blood pressure ≥140 mmHg or diastolic blood pressure ≥90 mmHg for the last BP measurement prior to December 31 2019. The BP treatment target was chosen to align with the Hypertension Canada’s 2016 Canadian hypertension education program guidelines for blood pressure measurement, diagnosis, assessment of risk, prevention, and treatment of hypertension that reflected the practice during the study period.^23^ The SBP treatment goal is a pressure level of <140 mm Hg (Grade C). The DBP treatment goal is a pressure level of <90 mm Hg (Grade A). These targets were established using Office-based BP Measurements (OBPM).^23^ For patients with multiple BP readings recorded on the same day at the same visit (eg, using an automated BPtru machine), we took the average measurement of the systolic and diastolic blood pressure measures on that day.

We also conducted a sensitivity analysis, with a BP target of <130/80 mmHg, as recommended by the 2018 ESC/ESH Guidelines for the management of arterial hypertension,^24^ and the 2017 ACC/AHA/AAPA/ABC/ACPM/AGS/APhA/ASH/ASPC/NMA/PCNA Guideline for the Prevention, Detection, Evaluation, and Management of High Blood Pressure in Adults.^25^

### Statistical Analysis

We used descriptive statistics to summarize patient demographics and clinical characteristics associated with controlled and uncontrolled blood pressure. We used means, standard deviations, and median, minimum and maximum to summarize continuous variables, while frequencies and percentages were used to summarize categorical variables. To reduce the risk of re-identification, our reports exclude any cell count of 5 or fewer. Comparisons between patients with controlled and uncontrolled BP were assessed using Pearson’s chi-square tests for categorical covariates and using 2-sample *t*-tests for continuous covariates.

We fitted logistic regression models using the Generalized Estimating Equations (GEE) with an exchangeable working correlation matrix to assess the association between multimorbidity (or individual long-term condition) on uncontrolled BP. We reported both the unadjusted odds ratios (OR) and adjusted odds ratios. To account for multicollinearity, we fitted separate logistic models using GEE for multimorbidity and different comorbidities, respectively. We a priori specified age, sex, income quintiles, smoking, and BMI as potential confounders (ie, common cause) for multimorbidity and for the outcome of BP control, while different classes of hypertension medications were considered potential mediators on the pathway between multimorbidity and BP control (see Supplemental Figure 1). In GEE models, we adjusted for the following covariates: age, sex, income quintiles, smoking status, BMI, number of primary care visits, along with the multimorbidity status.

In a sensitivity analysis, we used a reduced threshold for blood pressure control (<130/80 mm Hg) and excluded BMI and smoking status in the regression models to include patients that did not have BMI or smoking status recorded.

### Ethical Approval

This study was approved by the Research Ethics Board at the University of Toronto (protocol number 00043354).

## Results

### Sample Characteristics

A total of 67 385 patients who satisfied the selection criteria were included in this study. Patients in the sample had a mean age of 70.1 years (SD = 11.8) and 53.1% were female. The prevalence of overweight and obesity was 66%, with a greater proportion of patients with class II and class III obesity in the uncontrolled BP group compared to the controlled BP group. There was no difference in socioeconomic status between the 2 groups (Table 1).

**Table 1.** Patient Characteristics.

	Controlled BP (N = 43 303)	Uncontrolled BP (N = 24 082)	Overall (N = 67 385)
Age (years)			
Mean (SD)	70.3 (11.6)	69.6 (12.2)	70.1 (11.8)
Median [min, max]	70.0 [47.0, 111]	69.0 [47.0, 110]	70.0 [47.0, 111]
Age group (years)			
45-74	30 562 (70.6%)	17 256 (71.7%)	47 818 (71.0%)
75-84	8927 (20.6%)	4486 (18.6%)	13 413 (19.9%)
85+	3814 (8.8%)	2340 (9.7%)	6154 (9.1%)
Sex			
Female	22 637 (52.3%)	13 132 (54.5%)	35 769 (53.1%)
Male	20 666 (47.7%)	10 950 (45.5%)	31 616 (46.9%)
Income quintiles			
1 (low income)	8984 (20.7%)	5090 (21.1%)	14 074 (20.9%)
2	7812 (18.0%)	4328 (18.0%)	12 140 (18.0%)
3	7264 (16.8%)	3938 (16.4%)	11 202 (16.6%)
4	7572 (17.5%)	4177 (17.3%)	11 749 (17.4%)
5 (high income)	10 693 (24.7%)	5976 (24.8%)	16 669 (24.7%)
Missing	978 (2.3%)	573 (2.4%)	1551 (2.3%)
Multimorbidity			
No	7473 (17.3%)	5574 (23.1%)	13 047 (19.4%)
Yes	35 830 (82.7%)	18 508 (76.9%)	54 338 (80.6%)
Body mass index level			
Missing	7831 (18.1%)	5018 (20.8%)	12 849 (19.1%)
18.4 or less (underweight)	267 (0.6%)	145 (0.6%)	412 (0.6%)
18.5-24.9 (normal)	6453 (14.9%)	3220 (13.4%)	9673 (14.4%)
25-29.9 (overweight)	13 470 (31.1%)	6999 (29.1%)	20 469 (30.4%)
30-34.9 (obese Class I)	9278 (21.4%)	5095 (21.2%)	14 373 (21.3%)
35-39.9 (obese Class II)	3782 (8.7%)	2208 (9.2%)	5990 (8.9%)
40 or more (obese Class III)	2222 (5.1%)	1397 (5.8%)	3619 (5.4%)
Most recent smoking status			
Missing	11 062 (25.5%)	6740 (28.0%)	17 802 (26.4%)
Current	4219 (9.7%)	2542 (10.6%)	6761 (10.0%)
Non-smoker	19 999 (46.2%)	10 868 (45.1%)	30 867 (45.8%)
Past	8023 (18.5%)	3932 (16.3%)	11 955 (17.7%)
Cancer			
No	37 881 (87.5%)	21 349 (88.7%)	59 230 (87.9%)
Yes	5422 (12.5%)	2733 (11.3%)	8155 (12.1%)
Ischemic heart disease			
No	37 182 (85.9%)	21 679 (90.0%)	58 861 (87.4%)
Yes	6121 (14.1%)	2403 (10.0%)	8524 (12.6%)
Heart failure			
No	40 754 (94.1%)	23 082 (95.8%)	63 836 (94.7%)
Yes	2549 (5.9%)	1000 (4.2%)	3549 (5.3%)
Atrial fibrillation			
No	38 362 (88.6%)	21 878 (90.8%)	60 240 (89.4%)
Yes	4941 (11.4%)	2204 (9.2%)	7145 (10.6%)
Diabetes mellitus			
No	28 126 (65.0%)	17 210 (71.5%)	45 336 (67.3%)
Yes	15 177 (35.0%)	6872 (28.5%)	22 049 (32.7%)
Chronic kidney disease			
No	35 292 (81.5%)	20 021 (83.1%)	55 313 (82.1%)
Yes	8011 (18.5%)	4061 (16.9%)	12 072 (17.9%)
Chronic obstructive pulmonary disease			
No	39 365 (90.9%)	22 197 (92.2%)	61 562 (91.4%)
Yes	3938 (9.1%)	1885 (7.8%)	5823 (8.6%)
Osteoarthritis			
No	26 654 (61.6%)	15 500 (64.4%)	42 154 (62.6%)
Yes	16 649 (38.4%)	8582 (35.6%)	25 231 (37.4%)
Dementia			
No	39 774 (91.9%)	22 422 (93.1%)	62 196 (92.3%)
Yes	3529 (8.1%)	1660 (6.9%)	5189 (7.7%)
Depression and/or anxiety			
No	28 943 (66.8%)	16 728 (69.5%)	45 671 (67.8%)
Yes	14 360 (33.2%)	7354 (30.5%)	21 714 (32.2%)
Schizophrenia			
No	42 726 (98.7%)	23 853 (99.0%)	66 579 (98.8%)
Yes	577 (1.3%)	229 (1.0%)	806 (1.2%)

Comparisons between patients with controlled and uncontrolled BP were assessed using Pearson’s chi-square tests for categorical covariates and 2-sample *t*-tests for continuous covariates. All *P* < .001.

### Multimorbidity

The overall prevalence of multimorbidity was 80.6%. Patients with multimorbidity had a higher number of annual primary care visits than patients without multimorbidity in 2017, 2018, and 2019 (Supplemental Table 2). As an example, 8.0% of the patients with multimorbidity had more than 9 visits in 2019 compared to 1.9% of patients without multimorbidity

### Uncontrolled Blood Pressure

**Figure 2.**
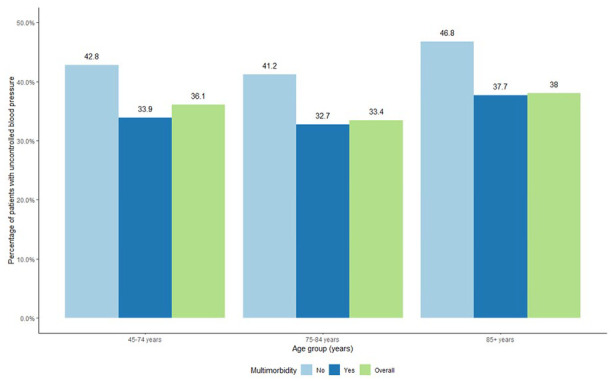
Percentage of patients with uncontrolled blood pressure.

Overall, 35.7% of patients had uncontrolled BP (34.1% in patients with multimorbidity and 42.7% in patients without multimorbidity, *P* < .001). Figure 2 presents the percentages of uncontrolled BP in patients with and without multimorbidity. Across the 3 age groups, patients with multimorbidity had lower rates of uncontrolled BP compared to patients without multimorbidity.

### The Association Between Multimorbidity and Hypertension Control

**Figure 3.**
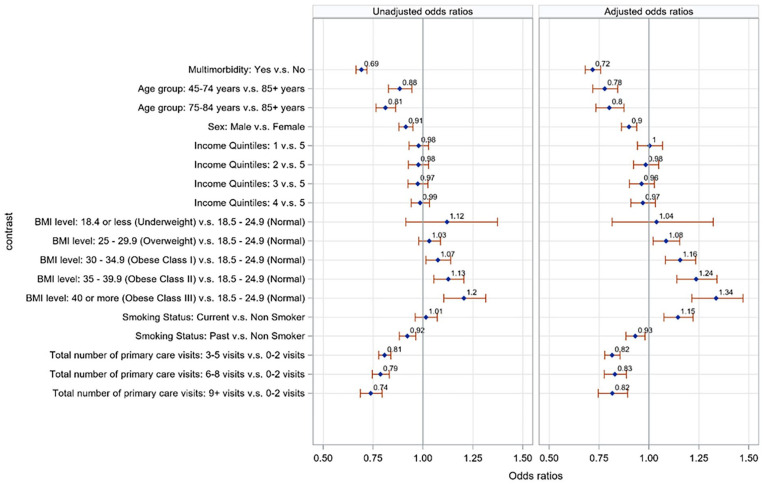
Unadjusted and adjusted odds ratios for uncontrolled blood pressure with respect to patient characteristics. *Odds ratio >1 means a greater likelihood of uncontrolled blood pressure. Models were adjusted for age, sex, income quintiles, smoking status, body mass index, and number of visits.

Patients with multimorbidity had lower odds of uncontrolled BP compared to those without multimorbidity (adjusted OR = 0.72, 95%CI 0.68-0.76, adjusted for age, sex, income quintiles, smoking status, body mass index, and number of visits). Younger patients had lower odds of having uncontrolled BP than older patients. Obese patients (in class I, II, and III) had higher odds of uncontrolled BP than those with normal BMI. Current smokers had higher odds of having uncontrolled BP compared to non-smokers. Increasing number of visits to primary care doctors was associated with lower odds of uncontrolled BP (Figure 3).

### The Association Between Types of Long-Term Conditions and Hypertension Control

**Figure 4.**
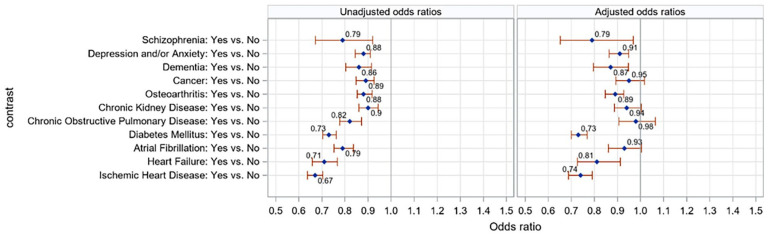
Unadjusted and adjusted odds ratios for uncontrolled blood pressure with respect to individual long-term conditions. *Odds ratio >1 means a greater likelihood of uncontrolled blood pressure. Models were adjusted for age, sex, income quintiles, smoking status, body mass index, and number of visits.

Among the long-term conditions, 7 conditions were significantly associated with lower likelihood of uncontrolled BP: diabetes (adjusted OR = 0.73, 95%CI 0.70-0.77), heart failure (adjusted OR = 0.81, 95%CI 0.73-0.91), ischemic heart disease (adjusted OR = 0.74, 95%CI 0.69-0.79), schizophrenia (adjusted OR = 0.79, 95%CI 0.65-0.97), depression/anxiety (adjusted OR = 0.91, 95%CI 0.86-0.95), dementia (adjusted OR = 0.87, 95%CI 0.80-0.95), and osteoarthritis (adjusted OR = 0.89, 95%CI 0.85-0.93). The remaining long-term conditions (atrial fibrillation, chronic obstructive pulmonary disease, chronic kidney disease, and cancer) were not associated with uncontrolled BP (Figure 4).

### Sensitivity Analysis

Sensitivity analyses showed consistent results (Supplemental Tables 3 and 4).

In GEE models using ≥130/80 mmHg as the threshold for sub-optimal blood pressure control, multimorbidity was still significantly associated with reduced likelihoods of uncontrolled BP (adjusted ORs 0.56, 95%CI 0.54-0.59), adjusting for age, sex, income quintiles, body mass index, and number of visits.

## Discussion

Our analysis in 67 385 Canadians aged 45 years or more with hypertension attending primary care from 2017 to 2019 showed that multimorbidity was associated with approximately 30% lower odds of uncontrolled BP. The individual long-term conditions that were significantly associated with lower likelihood of uncontrolled BP include diabetes, heart failure, ischemic heart disease, schizophrenia, depression/anxiety, dementia, or osteoarthritis.

Our finding aligns with other studies that examined this association, which reported that multimorbidity was one of the strongest predictors of controlled BP in patients with hypertension. Tapela et al^26^ found that multimorbidity was positively associated with BP control among participants with hypertension in the UK Biobank, and the types of comorbidities that were significantly associated with controlled BP included cardiovascular disease (OR = 2.11, 95%CI 2.04-2.19), diabetes (OR = 1.32, 95%CI 1.27-1.36), migraines (OR = 1.68, 95%CI 1.56-1.81), and depression (OR = 1.27, 95%CI 1.20-1.34). Another study conducted by Sarkar et al^12^ in 31 676 patients with hypertension in primary care in the UK, found that 16 140 (51 %) had multimorbidity, and hypertensive patients with multimorbidity had lower BP than those with hypertension alone. Mini et al^16^ also reported that blood pressure control was better in participants who reported any comorbidity (OR = 2.37, 95%CI 1.51-3.71) compared to those who did not report any. However, several other studies reported the opposite findings—people with more comorbidities had poorer management and control of hypertension. In a study of 223 286 patients with hypertension in Hongkong, Wong et al^15^ found that the proportion of patients having poor BP control increased from 35.0% to 65.0% and 69.1% when the number of medical conditions increased from 0 to 1 and 2, respectively. However, this study enrolled adult patients prescribed their first antihypertensive agents; hence people with comorbidities (such as diabetes, heart failure, and coronary heart disease) who were already on a relevant medication (beta-blockers, ACE inhibitors) for these reasons would be excluded from the hypertension cohort. A cross-sectional analysis of the World Health Organization Study of Global Ageing and Adult Health (WHO SAGE) Wave 1 (2007-2010) in 41 557 adults (9778 with hypertension) from 6 middle-income countries (including China, Ghana, India, Mexico, Russia, and South Africa) reported that more comorbidities were associated with increased odds of uncontrolled hypertension.^17^

A possible explanation that would account for the lower odds of uncontrolled hypertension in patients with chronic conditions like diabetes, heart failure, ischemic heart disease, schizophrenia, depression/anxiety, or osteoarthritis may be explained by the fact that patients with these conditions tend to have frequent doctor visits. As with dementia, some studies showed that dementia was associated with lower blood pressure.^27,28^

The results from this study may reflect the achievement of primary care in controlling cardiovascular risk factors in patients with multimorbidity, including regular BP measures and treatment. Comorbidities may be associated with more frequent healthcare utilization and hence increase the monitoring of chronic conditions, including BP measurement and counselling on lifestyle modifications and medication adherence. Another explanation could be the study’s participation selection processes. Among people with hypertension as their only chronic health condition, those with poorer BP control may be more motivated to attend primary care and hence were included in this cohort. Thus, the observed associations could be explained by selection bias—hypertensive patients with no comorbidities, but uncontrolled BP may have been more likely to seek primary care compared to hypertensive patients with no comorbidities and controlled BP. These lead to opportunities to measure BP and obtain scripts, reminders given to them on taking medications, and better lifestyle modifications.

### Strengths

This study has several strengths. To the best of our knowledge, it is the first study reporting the association between multimorbidity and blood pressure control in Canada. The study is based on detailed clinical data. It is grounded in real world data from routine primary care practices. The large sample size of this study provides sufficient statistical power, allowing for point estimates with precise confidence intervals.

### Limitations

The study data was only collected from patients in Ontario and is not nationally representative. We might be unable to capture all cases of hypertension and/or prescriptions of anti-hypertensive medications in the EMR data. Duration and severity of hypertension, and lifestyle modification, an essential strategy in the management of hypertension and can influence BP control, could not be captured through the EMR data. In addition, BP control was defined based on BP measurement at the clinics, which may be influenced by the white coat effect. Therefore, results should be replicated and cautiously interpreted before generalizing to all patients in primary care.

## Conclusion

In this large-scale study of patients attending primary care with hypertension, multimorbidity was associated with better BP control. Several long-term conditions were associated with better control, including diabetes, heart failure, ischemic heart disease, schizophrenia, depression/anxiety, dementia, and osteoarthritis. Further large‑scale studies are needed to explore the mechanisms underlying the associations between multimorbidity and BP control, and to understand barriers to BP control in primary care.

## Supplemental Material

sj-docx-1-jpc-10.1177_21501319231215025 – Supplemental material for Multimorbidity and Blood Pressure Control in Patients Attending Primary Care in Canada Click here for additional data file. Supplemental material, sj-docx-1-jpc-10.1177_21501319231215025 for Multimorbidity and Blood Pressure Control in Patients Attending Primary Care in Canada by Tu N. Nguyen, Sumeet Kalia, Peter Hanlon, Bhautesh D. Jani, Barbara I. Nicholl, Chelsea D. Christie, Babak Aliarzadeh, Rahim Moineddin, Christopher Harrison, Clara Chow, Martin Fortin, Frances S. Mair and Michelle Greiver in Journal of Primary Care & Community Health
