# Supplementary material for: Multimorbidity and Blood Pressure Control in Patients Attending Primary Care in Canada
Source: J Prim Care Community Health. 2023 Dec 14;14:21501319231215025. doi: 10.1177/21501319231215025 (PMC10725138; doi:10.1177/21501319231215025)
Supplement: sj-docx-1-jpc-10.1177_21501319231215025 – Supplemental material for Multimorbidity and Blood Pressure Control in Patients Attending Primary Care in Canada [file sj-docx-1-jpc-10.1177_21501319231215025.docx]

**Supplementary data**

**Figure S1: Directed acyclic graph for the effect of multi-morbidity on blood pressure control**


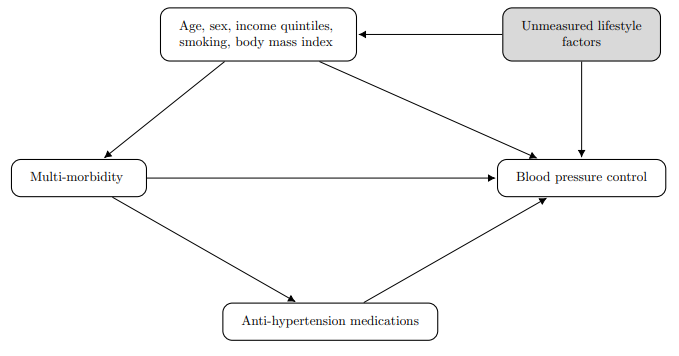


**Table S1: Unadjusted and adjusted odds ratios for uncontrolled blood pressure with respect to patient characteristics**

| Type | Contrast | Odds ratios | Upper | Lower | P-value |
| --- | --- | --- | --- | --- | --- |
| Unadjusted odds ratios | Total number of primary care visits: 9+ visits v.s. 0-2 visits | 0.73827 | 0.79546 | 0.68520 | <0.001 |
| Unadjusted odds ratios | Total number of primary care visits: 6-8 visits v.s. 0-2 visits | 0.78719 | 0.83093 | 0.74576 | <0.001 |
| Unadjusted odds ratios | Total number of primary care visits: 3-5 visits v.s. 0-2 visits | 0.80753 | 0.83877 | 0.77746 | <0.001 |
| Unadjusted odds ratios | Smoking Status: Past v.s. Non Smoker | 0.92141 | 0.96352 | 0.88113 | <0.001 |
| Unadjusted odds ratios | Smoking Status: Current v.s. Non Smoker | 1.01420 | 1.07104 | 0.96038 | 0.612 |
| Unadjusted odds ratios | BMI level: 40 or more (Obese Class III) v.s. 18.5 - 24.9 (Normal) | 1.20424 | 1.31402 | 1.10364 | <0.001 |
| Unadjusted odds ratios | BMI level: 35 - 39.9 (Obese Class II) v.s. 18.5 - 24.9 (Normal) | 1.12685 | 1.20470 | 1.05403 | <0.001 |
| Unadjusted odds ratios | BMI level: 30 - 34.9 (Obese Class I) v.s. 18.5 - 24.9 (Normal) | 1.07457 | 1.13840 | 1.01432 | 0.015 |
| Unadjusted odds ratios | BMI level: 25 - 29.9 (Overweight) v.s. 18.5 - 24.9 (Normal) | 1.03133 | 1.08717 | 0.97836 | 0.251 |
| Unadjusted odds ratios | BMI level: 18.4 or less (Underweight) v.s. 18.5 - 24.9 (Normal) | 1.11997 | 1.37281 | 0.91370 | 0.275 |
| Unadjusted odds ratios | Income Quintiles: 4 v.s. 5 | 0.98538 | 1.03246 | 0.94044 | 0.536 |
| Unadjusted odds ratios | Income Quintiles: 3 v.s. 5 | 0.97333 | 1.02475 | 0.92448 | 0.303 |
| Unadjusted odds ratios | Income Quintiles: 2 v.s. 5 | 0.97613 | 1.02786 | 0.92701 | 0.359 |
| Unadjusted odds ratios | Income Quintiles: 1 v.s. 5 | 0.97758 | 1.02763 | 0.92997 | 0.373 |
| Unadjusted odds ratios | Sex: Male v.s. Female | 0.91376 | 0.94975 | 0.87915 | <0.001 |
| Unadjusted odds ratios | Age group: 75-84 years v.s. 85+ years | 0.81139 | 0.86227 | 0.76352 | <0.001 |
| Unadjusted odds ratios | Age group: 45-74 years v.s. 85+ years | 0.88376 | 0.94439 | 0.82703 | <0.001 |
| Unadjusted odds ratios | Multimorbidity: Yes v.s. No | 0.69121 | 0.71972 | 0.66384 | <0.001 |
| Adjusted odds ratios | Total number of primary care visits: 9+ visits v.s. 0-2 visits | 0.81565 | 0.89297 | 0.74502 | <0.001 |
| Adjusted odds ratios | Total number of primary care visits: 6-8 visits v.s. 0-2 visits | 0.82926 | 0.88645 | 0.77576 | <0.001 |
| Adjusted odds ratios | Total number of primary care visits: 3-5 visits v.s. 0-2 visits | 0.81522 | 0.85468 | 0.77759 | <0.001 |
| Adjusted odds ratios | Smoking Status: Past v.s. Non Smoker | 0.93085 | 0.97948 | 0.88463 | 0.006 |
| Adjusted odds ratios | Smoking Status: Current v.s. Non Smoker | 1.14532 | 1.22043 | 1.07484 | <0.001 |
| Adjusted odds ratios | BMI level: 40 or more (Obese Class III) v.s. 18.5 - 24.9 (Normal) | 1.33611 | 1.47065 | 1.21388 | <0.001 |
| Adjusted odds ratios | BMI level: 35 - 39.9 (Obese Class II) v.s. 18.5 - 24.9 (Normal) | 1.23587 | 1.34055 | 1.13938 | <0.001 |
| Adjusted odds ratios | BMI level: 30 - 34.9 (Obese Class I) v.s. 18.5 - 24.9 (Normal) | 1.15571 | 1.23387 | 1.08249 | <0.001 |
| Adjusted odds ratios | BMI level: 25 - 29.9 (Overweight) v.s. 18.5 - 24.9 (Normal) | 1.08476 | 1.15337 | 1.02024 | 0.009 |
| Adjusted odds ratios | BMI level: 18.4 or less (Underweight) v.s. 18.5 - 24.9 (Normal) | 1.03778 | 1.32207 | 0.81462 | 0.764 |
| Adjusted odds ratios | Income Quintiles: 4 v.s. 5 | 0.96900 | 1.03199 | 0.90985 | 0.327 |
| Adjusted odds ratios | Income Quintiles: 3 v.s. 5 | 0.96202 | 1.02630 | 0.90176 | 0.241 |
| Adjusted odds ratios | Income Quintiles: 2 v.s. 5 | 0.98320 | 1.04831 | 0.92213 | 0.604 |
| Adjusted odds ratios | Income Quintiles: 1 v.s. 5 | 1.00317 | 1.06821 | 0.94208 | 0.921 |
| Adjusted odds ratios | Sex: Male v.s. Female | 0.89949 | 0.93914 | 0.86152 | <0.001 |
| Adjusted odds ratios | Age group: 75-84 years v.s. 85+ years | 0.80118 | 0.87470 | 0.73384 | <0.001 |
| Adjusted odds ratios | Age group: 45-74 years v.s. 85+ years | 0.77803 | 0.84269 | 0.71832 | <0.001 |
| Adjusted odds ratios | Multimorbidity: Yes v.s. No | 0.71761 | 0.75742 | 0.67990 | <0.001 |

**Table S2: Mean number of primary care visits for patients with and without multi-morbidity (annually)**

| Mean (standard deviation) number of primary care visits annually | multimorbidity | |
| --- | --- | --- |
|  | **No** | **Yes** |
| 2017 | 2.14 (2.17) | 3.80 (3.44) |
| 2018 | 2.30 (2.22) | 3.88 (3.36) |
| 2019 | 2.44 (2.19) | 3.91 (3.28) |
| Frequency (N, column %) | | |
| 2017 | | |
| 0-2 visits | \| 8599 \| \| --- \| \| 65.91 \| | \| 22134 \| \| --- \| \| 40.73 \| |
| 3-5 visits | \| 3523 \| \| --- \| \| 27.00 \| | \| 19794 \| \| --- \| \| 36.43 \| |
| 6-8 visits | \| 729 \| \| --- \| \| 5.59 \| | \| 7838 \| \| --- \| \| 14.42 \| |
| 9+ visits | \| 196 \| \| --- \| \| 1.50 \| | \| 4572 \| \| --- \| \| 8.41 \| |
| 2018 | | |
| 0-2 visits | \| 8219 \| \| --- \| \| 63.00 \| | \| 21141 \| \| --- \| \| 38.91 \| |
| 3-5 visits | \| 3813 \| \| --- \| \| 29.23 \| | \| 20635 \| \| --- \| \| 37.98 \| |
| 6-8 visits | \| 779 \| \| --- \| \| 5.97 \| | \| 8143 \| \| --- \| \| 14.99 \| |
| 9+ visits | \| 236 \| \| --- \| \| 1.81 \| | \| 4419 \| \| --- \| \| 8.13 \| |
| 2019 | | |
| 0-2 visits | \| 7934 \| \| --- \| \| 60.81 \| | \| 20688 \| \| --- \| \| 38.07 \| |
| 3-5 visits | \| 4024 \| \| --- \| \| 30.84 \| | \| 21156 \| \| --- \| \| 38.93 \| |
| 6-8 visits | \| 846 \| \| --- \| \| 6.48 \| | \| 8145 \| \| --- \| \| 14.99 \| |
| 9+ visits | \| 243 \| \| --- \| \| 1.86 \| | \| 4349 \| \| --- \| \| 8.00 \| |

**Table S3: Patient characteristics for sub-optimal blood pressure control (≥130/80 mmHg)**

|  | **Controlled BP (N=19535)** | **Uncontrolled BP (N=47850)** | **Overall (N=67385)** | **P-value** |
| --- | --- | --- | --- | --- |
| **Age (years)** |  |  |  |  |
| Mean (SD) | 72.1 (11.5) | 69.2 (11.9) | 70.1 (11.8) | <0.001 |
| Median [Min, Max] | 72.0 [47.0, 111] | 69.0 [47.0, 110] | 70.0 [47.0, 111] |  |
| **Age group (years)** |  |  |  |  |
| 45-74 years | 12809 (65.6%) | 35009 (73.2%) | 47818 (71.0%) | <0.001 |
| 75-84 years | 4597 (23.5%) | 8816 (18.4%) | 13413 (19.9%) |  |
| 85+ years | 2129 (10.9%) | 4025 (8.4%) | 6154 (9.1%) |  |
| **Sex** |  |  |  |  |
| Female | 10183 (52.1%) | 25586 (53.5%) | 35769 (53.1%) | 0.00652 |
| Male | 9352 (47.9%) | 22264 (46.5%) | 31616 (46.9%) |  |
| **Income Quintiles** |  |  |  |  |
| 1 (low income) | 4259 (21.8%) | 9815 (20.5%) | 14074 (20.9%) | <0.001 |
| 2 | 3605 (18.5%) | 8535 (17.8%) | 12140 (18.0%) |  |
| 3 | 3223 (16.5%) | 7979 (16.7%) | 11202 (16.6%) |  |
| 4 | 3323 (17.0%) | 8426 (17.6%) | 11749 (17.4%) |  |
| 5 (high income) | 4626 (23.7%) | 12043 (25.2%) | 16669 (24.7%) |  |
| Missing | 499 (2.6%) | 1052 (2.2%) | 1551 (2.3%) |  |
| **Multimorbidity** |  |  |  |  |
| No | 2528 (12.9%) | 10519 (22.0%) | 13047 (19.4%) | <0.001 |
| Yes | 17007 (87.1%) | 37331 (78.0%) | 54338 (80.6%) |  |
| **Body mass index level** |  |  |  |  |
| Missing | 3347 (17.1%) | 9502 (19.9%) | 12849 (19.1%) | <0.001 |
| 18.4 or less (Underweight) | 152 (0.8%) | 260 (0.5%) | 412 (0.6%) |  |
| 18.5 - 24.9 (Normal) | 3226 (16.5%) | 6447 (13.5%) | 9673 (14.4%) |  |
| 25 - 29.9 (Overweight) | 6190 (31.7%) | 14279 (29.8%) | 20469 (30.4%) |  |
| 30 - 34.9 (Obese Class I) | 4043 (20.7%) | 10330 (21.6%) | 14373 (21.3%) |  |
| 35 - 39.9 (Obese Class II) | 1630 (8.3%) | 4360 (9.1%) | 5990 (8.9%) |  |
| 40 or more (Obese Class III) | 947 (4.8%) | 2672 (5.6%) | 3619 (5.4%) |  |
| **Most recent smoking status** |  |  |  |  |
| Missing | 4935 (25.3%) | 12867 (26.9%) | 17802 (26.4%) | <0.001 |
| Current | 1909 (9.8%) | 4852 (10.1%) | 6761 (10.0%) |  |
| Non Smoker | 8856 (45.3%) | 22011 (46.0%) | 30867 (45.8%) |  |
| Past | 3835 (19.6%) | 8120 (17.0%) | 11955 (17.7%) |  |
| **Cancer** |  |  |  |  |
| No | 16955 (86.8%) | 42275 (88.3%) | 59230 (87.9%) | <0.001 |
| Yes | 2580 (13.2%) | 5575 (11.7%) | 8155 (12.1%) |  |
| **Ischemic Heart disease** |  |  |  |  |
| No | 16032 (82.1%) | 42829 (89.5%) | 58861 (87.4%) | <0.001 |
| Yes | 3503 (17.9%) | 5021 (10.5%) | 8524 (12.6%) |  |
| **Heart failure** |  |  |  |  |
| No | 18006 (92.2%) | 45830 (95.8%) | 63836 (94.7%) | <0.001 |
| Yes | 1529 (7.8%) | 2020 (4.2%) | 3549 (5.3%) |  |
| **Atrial fibrillation** |  |  |  |  |
| No | 16799 (86.0%) | 43441 (90.8%) | 60240 (89.4%) | <0.001 |
| Yes | 2736 (14.0%) | 4409 (9.2%) | 7145 (10.6%) |  |
| **Diabetes Mellitus** |  |  |  |  |
| No | 11842 (60.6%) | 33494 (70.0%) | 45336 (67.3%) | <0.001 |
| Yes | 7693 (39.4%) | 14356 (30.0%) | 22049 (32.7%) |  |
| **Chronic Kidney disease** |  |  |  |  |
| No | 15193 (77.8%) | 40120 (83.8%) | 55313 (82.1%) | <0.001 |
| Yes | 4342 (22.2%) | 7730 (16.2%) | 12072 (17.9%) |  |
| **Chronic Obstructive Pulmonary Disease** |  |  |  |  |
| No | 17487 (89.5%) | 44075 (92.1%) | 61562 (91.4%) | <0.001 |
| Yes | 2048 (10.5%) | 3775 (7.9%) | 5823 (8.6%) |  |
| **Mental health** |  |  |  |  |
| No | 11804 (60.4%) | 30997 (64.8%) | 42801 (63.5%) | <0.001 |
| Yes | 7731 (39.6%) | 16853 (35.2%) | 24584 (36.5%) |  |

**Table S4: Adjusted odds ratios using GEE model for sensitivity analysis using ≥ 130/80 mmHg as the threshold for sub-optimal blood pressure control.**

| Contrast | Adjusted odds ratios | Lower | Upper | P-value |
| --- | --- | --- | --- | --- |
| Multimorbidity: Yes vs. No | 0.56 | 0.54 | 0.59 | <0.001 |
| Age group: 45-74 years vs. 85+ years | 1.29 | 1.22 | 1.37 | <0.001 |
| Age group: 75-84 years vs. 85+ years | 0.98 | 0.92 | 1.05 | 0.582 |
| Sex: Male vs. Female | 0.91 | 0.88 | 0.94 | <0.001 |
| Income Quintiles: 1 vs. 5 | 0.93 | 0.88 | 0.98 | 0.005 |
| Income Quintiles: 2 vs. 5 | 0.95 | 0.90 | 1.00 | 0.062 |
| Income Quintiles: 3 vs. 5 | 0.97 | 0.92 | 1.02 | 0.34 |
| Income Quintiles: 4 vs. 5 | 0.98 | 0.93 | 1.03 | 0.405 |
| Region: Urban vs. Rural | 0.84 | 0.78 | 0.90 | <0.001 |

**Table S5: Unadjusted and adjusted odds ratios for uncontrolled blood pressure with respect to co-morbidities**

| Type | Contrast | Odd ratios | Lower | Upper | P-Value |
| --- | --- | --- | --- | --- | --- |
| Unadjusted odds ratios | Ischemic Heart Disease: Yes vs. No | 0.66969 | 0.63707 | 0.70399 | <0.001 |
| Unadjusted odds ratios | Heart Failure: Yes vs. No | 0.71025 | 0.65731 | 0.76745 | <0.001 |
| Unadjusted odds ratios | Atrial Fibrillation: Yes vs. No | 0.79316 | 0.75169 | 0.83692 | <0.001 |
| Unadjusted odds ratios | Diabetes Mellitus: Yes vs. No | 0.73233 | 0.70360 | 0.76223 | <0.001 |
| Unadjusted odds ratios | Chronic Obstructive Pulmonary Disease: Yes vs. No | 0.82250 | 0.77670 | 0.87100 | <0.001 |
| Unadjusted odds ratios | Chronic Kidney Disease: Yes vs. No | 0.90107 | 0.86078 | 0.94326 | <0.001 |
| Unadjusted odds ratios | Osteoarthritis: Yes vs. No | 0.88485 | 0.85331 | 0.91755 | <0.001 |
| Unadjusted odds ratios | Cancer: Yes vs. No | 0.88660 | 0.84844 | 0.92646 | <0.001 |
| Unadjusted odds ratios | Dementia: Yes vs. No | 0.85688 | 0.80274 | 0.91466 | <0.001 |
| Unadjusted odds ratios | Depression and/or Anxiety: Yes vs. No | 0.87679 | 0.84454 | 0.91028 | <0.001 |
| Unadjusted odds ratios | Schizophrenia: Yes vs. No | 0.78581 | 0.67154 | 0.91952 | <0.001 |
| Adjusted odds ratios | Ischemic Heart Disease: Yes vs. No | 0.73727 | 0.68770 | 0.79041 | <0.001 |
| Adjusted odds ratios | Heart Failure: Yes vs. No | 0.81404 | 0.72606 | 0.91268 | <0.001 |
| Adjusted odds ratios | Atrial Fibrillation: Yes vs. No | 0.92968 | 0.86071 | 1.00417 | 0.064 |
| Adjusted odds ratios | Diabetes Mellitus: Yes vs. No | 0.73368 | 0.70013 | 0.76885 | <0.001 |
| Adjusted odds ratios | Chronic Obstructive Pulmonary Disease: Yes vs. No | 0.98163 | 0.90611 | 1.06344 | 0.65 |
| Adjusted odds ratios | Chronic Kidney Disease: Yes vs. No | 0.94286 | 0.88606 | 1.00330 | 0.063 |
| Adjusted odds ratios | Osteoarthritis: Yes vs. No | 0.88625 | 0.84715 | 0.92716 | <0.001 |
| Adjusted odds ratios | Cancer: Yes vs. No | 0.95232 | 0.89199 | 1.01672 | 0.143 |
| Adjusted odds ratios | Dementia: Yes vs. No | 0.86759 | 0.79505 | 0.94675 | 0.001 |
| Adjusted odds ratios | Depression and/or Anxiety: Yes vs. No | 0.90557 | 0.86453 | 0.94856 | <0.001 |
| Adjusted odds ratios | Schizophrenia: Yes vs. No | 0.79424 | 0.65170 | 0.96795 | 0.022 |

**Table S6: Sub-group analysis on physical and psychological multi-morbidity.**

*We added a sub-group analysis where the multi-morbidity is defined using a set of physical and psychological morbidities. Below, we present the results for the proportion of patients with a specific type of multi-morbidity (physical or psychological). The magnitude and direction of the odds ratios for both psychological and physical multimorbidity is consistent with the findings reported in the main text of the paper.*

|  | **Psychological multi-morbidity*** | **Physical multimorbidity*** |
| --- | --- | --- |
| **Patients with multi-morbidity (N, %)** | **24,584 (36.5%)** | **48,833 (72.4%)** |
| **Patients with uncontrolled BP (N, %)** | **8,310 (33.8%)** | **16,369 (33.5%)** |
| **Odds ratios (95% CI) for uncontrolled BP** | **0.71 (95% CI: 0.68 - 0.73)** | **0.88 (95% CI: 0.85 - 0.91)** |

**Table S7: Subgroup analysis *to assess whether the proportion of patients with uncontrolled BP differs by the number of primary care visits***

*We notice that the proportion of patients with uncontrolled BP decreased as the patients had more primary care visits, and that these decreases were similar for both multimorbidity types.*

|  | | **Proportion of patients with uncontrolled blood pressure** | |
| --- | --- | --- | --- |
| **Number of primary care visits** | **N patients** | **Psychological multi-morbidity*** | **Physical multimorbidity*** |
| **<=2 visits** | **28,622 (42.5%)** | **0.36** | **0.36** |
| **3-5 visits** | **25,180 (37.4%)** | **0.33** | **0.32** |
| **6-8 visits** | **8,991 (13.3%)** | **0.32** | **0.32** |
| **9+ visits** | **4,592 (6.8%)** | **0.31** | **0.31** |

**Appendix**

The appendix section contains the following sections:

1. Drug names for different classes of hypertension medications;
2. Definition for the hypertension phenotype;
3. Definition for primary care visit in UTOPIAN database;

**Hypertension medication classes**

The following search criteria were used to identify hypertension medication:

| **Drug class name** | **Search criteria** |
| --- | --- |
| **ACE inhibitors** | %medication(in=ACE,  search='benazepril\|Lotensin\|captopril\|Capoten\|Captotec\|Captril\|cilazapril\|Inhibace\|Inhibase\|enalapril\|Vasotec\|fosinopril\|Monopril\|lisinopril\|Zestril\|Prinivil\|perindopril\|Coversyl\|quinapril hcl\|Accupril\|Accupro\|ramipril\|Altace\|trandolapril\|Mavik'); |
| **ARB inhibitors** | %medication(in=ARB,  search='candesartan\|Atacand\|eprosartan\|Teveten\|irbesartan\|Avapro\|losartan\|Cozaar\|olmesartan\|Olmetec\|telmisartan\|Micardis\|valsartan\|Diovan'); |
| **Calcium channels** | %medication(in=Ca_channel,  search='amlodipine\|Norvasc\|diltiazem\|Cardizem\|Tiazac\|felodipine\|Renedil\|Plendil\|nicardipine\|Cardene\|nifedipine\|Adalat\|nimodipine\|Minotop\|verapamil\|Isoptin\|Veralan\|Veramil'); |
| **Beta-blockers** | %medication(in=beta_blockers,  search='acebutolol\|Monitan\|Sectral\|Rhotral\|atenolol\|Tenormin\|bisoprolol\|Monocor\|labetalol\|Trandate\|metoprolol\|Lopressor\|Toprol\|Betaloc\|nadolol\|Corgard\|oxprenolol\|Trasicor\|pindolol\|Visken\|propranolol\|Inderal\|Detensol'); |
| **Diuretics** | %medication(in=diuretics,  search='amiloride\|Midamor\|spironolactone\|Aldactone\|triamterene\|Neo Diurex\|chlortalidone\|Thiazide\|Hygroton\|hydrochlorothiazide\|Oretic\|Microzide\|Diuchlor H\|Esidrix\|Hydro Aquil\|Hydrodiuril\|Neo Codema\|Urozide\|Apo-Hydro\|indapamide\|Lozide'); |
| **Other classes of hypertension medication** | **************************  Others includes  - Renin Inhibitor  - Alpha-2 Adrenergic Receptor Agonist  - combination medications  ********************************;  %medication(in=others,  search=  'aliskiren\|Basilez\|clonidine\|Catapres\|Catapresan\|Kapvay\|Dixarit\|methyldopa\|Dopazide\|Methazide\|Doparil\|  benazepril & hydrochlorothiazide\|Lotensin\|  cilazapril & hydrochlorothiazide\|Inhibace Plus\|  enalapril & hydrochlorothiazide\|Vaseretic\|  ramipril & hydrochlorothiazide\|Altace HCT\|  lisinopril & hydrochlorothiazide\|Zestoretic\|Prinzide\|  perindopril & indapamide\|Coversyl Plus\|  perindopril & amlodipine\|Viacoram\|  quinapril & hydrochlorothiazide\|Accuretic\|  amlodipine & telmisartan\|Twynsta\|  azilsartan & chlortalidone\|Edarbyclor\|  eprosartan & hydrochlorothiazide\|Teveten Plus\|  candesartan & hydrochlorothiazide\|Atacand Plus\|  hydrochlorothiazide & irbesartan\|Avalide\|  hydrochlorothiazide & losartan\|Hyzaar\|  hydrochlorothiazide & olmesartan\|Benicar HCT\|Olmetec Plus\|  valsartan & hydrochlorothiazide\|Diovan HCT\|  losartan & hydrochlorothiazide\|Hyzaar\|  telmisartan & hydrochlorothiazide\|Micardis HCT\|Micardis Plus\|  valsartan & sacubitril\|Entresto\|  atenolol & chlortalidone\|Tenoretic\|  atenolol & hydrochlorothiazide\|  felodipine & metoprolol\|Logimat\|Mibloc\|Mobloc\|Logimax\|Forte\|Mobloc Forte\|  nadolol & bendroflumethiazide\|Corzide\|  pindolol & hydrochlorothiazide\|Viskazide\|  propranolol & hydrochlorothiazide\|Inderide\|  timolol & hydrochlorothiazide\|Timolide\|  felodipine & ramipril\|Inotens\|Unimest And Unitens\|  verapamil & trandolapril\|Tarka\|  chlortalidone & reserpine\|Regroton\|  methyldopa & hydrochlorothiazide\|Aldoclor\|Aldoril\|  clonidine & chlortalidone\|Clorpres\|Combipres\|  reserpine & hydrochlorothiazide\|Hydroserpine\|Hydropres\|Serpasil Esidrix\|  reserpine & hydrochlorothiazide\|hydralazine hcl Ser-Ap-ES\|Serpazide\|Uni Serp\|Hydrap-Es\|Diuretic-Ap-Es\|Marpres\|Serathide\|Unipres\|Serpex\|  metoprolol & hydrochlorothiazide\|Dutoprol\|Lopressor HCT\|  metoprolol & chlortalidone\|Lopressidone\|Logroton\|  amiloride & hydrochlorothiazide\|Amiloride HCTZ\|Amihydro\|Riva-Amilzide\|Amilazide\|Amilzide\|Atenidone\|Moduretic\|  spironolactone & hydrochlorothiazide\|Aldactazide\|Spirozide\|Spirozine\|  triamterene & hydrochlorothiazide\|Dyazide\|Maxzide\|Diazide\|Pro Triazide\|Triamzide\|Riva-Zide\|Triazide\|Hydro-Triam'); |

**Hypertension phenotype**

We defined the hypertension phenotype using the following criteria:

1. Free text documentation of hypertension in the past or present health condition section of the cumulative patient included at least one of the following terms:

Include Do not include

Hypertension Hypertensive response

Hypertensive Borderline

Htn Occular/ocular

Maternal

Gestation

White coat

Pulmonary

Pregnancy induced/PIH

1. Anti-hypertensive medication (listed above) was prescribed and an elevated blood pressure reading was recorded at any point in the EMR
2. elevated blood pressure reading is defined as systolic blood pressure >= 140 mmHg or diastolic blood pressure >= 90 mmHg

OR

1. Anti-hypertensive medication (listed above) was prescribed and a billing record with the diagnosis code for hypertension (401) was found at any point in the EMR

OR

1. A billing record with the diagnosis code for hypertension (401) was found and an elevated blood pressure reading was recorded at any point in the EMR
2. elevated blood pressure reading is defined as systolic blood pressure >= 140 mmHg or diastolic blood pressure >= 90 mmHg

**Definition for primary care visit in UTOPIAN database**

OHIP service codes billed during the study period (Jan 2017 to Dec 2019) were used to select family physician visits that occurred via telephone, video, or in-person. Billing records for eligible patients containing any of the following service codes were counted as family physician visits:

| Code | Description | Code | Description |
| --- | --- | --- | --- |
| A001 | minor assessment | K017 | periodic health visit-child aft. 2nd birthday |
| A002 | enhanced 18-month well baby visit | K022 | hiv prim care individ care 1/2 hr or major part |
| A003 | major assessment | K028 | sexually transmitted disease (std) counseling |
| A004 | general re-assessment | K030 | diabetic management fee |
| A007 | intermediate assessment | K032 | gp-specific neurocognitive assessment |
| A008 | mini assessment | K033 | counselling - 1 pt/yr/unit |
| A071 | complex medical specific re-assessment | K039 | smoking cessation follow-up visit |
| A131 | complex medical specific re-assessment | K130 | periodic health visit - adolescent |
| A134 | medical specific re-assessment | K131 | periodic health visit - adult aged 18 to 64 inclusive |
| A624 | medical specific re-assessment | K132 | periodic health visit - adult 65 years of age and older |
| A888 | partial assessment | K680 | substance abuse - extended assessment |
| A903 | pre-op assessment | P003 | obs.-prenatal care-gen.assess-major prenatal visit |
| A920 | medical management of early pregnancy, initial visit | P004 | obs.-prenatal care-minor prenatal assess.-subseq.prenat.vis. |
| K005 | primary mental health | P005 | antenatal health screen |
| K007 | ind. psychotherapy per half hour - gp | P008 | obs.-post-natal care in office |
| K013 | counselling-one or more people-per 1/2hr | K037 | fibromyalgia/chronic fatigue syndrome care |
| K080 | Minor assessment of patient by telephone or video | K081 | Intermediate assessment including psychotherapy by telephone or video |
| K082 | Psychotherapy, psychiatric or mental health counselling by telephone or video | K087 | Minor assessment of an uninsured by telephone or video |
| K088 | Intermediate assessment of an uninsured patient including psychotherapy by telephone or video | K089 | Psychotherapy, psychiatric or mental health counselling of an uninsured patient by telephone or video |
